# Grafting of 18β-Glycyrrhetinic Acid and Sialic Acid onto Chitosan to Produce a New Amphipathic Chitosan Derivative: Synthesis, Characterization, and Cytotoxicity

**DOI:** 10.3390/molecules26020452

**Published:** 2021-01-16

**Authors:** Wei-Yan Quan, Song-Zhi Kong, Si-Dong Li, Hua-Zhong Liu, Qian-Qian Ouyang, Yong-Mei Huang, Hui Luo

**Affiliations:** 1 Department of Applied Chemistry, School of Chemistry and Environmental Science, Guangdong Ocean University, Zhanjiang 524088, China; quanfei183@126.com (W.-Y.Q.); 13702737491@163.com (S.-D.L.); liuhzbs@163.com (H.-Z.L.); 2 Marine Biomedical Research Institute, Guangdong Medical University, Zhanjiang 524023, China; ouyangqianqian0426@163.com (Q.-Q.O.); huangym@gdmu.edu.cn (Y.-M.H.); 3 Marine Biomedical Research Institute of Guangdong Zhanjiang, Zhanjiang 524023, China

**Keywords:** chitosan, chitosan derivatives, 18β-glycyrrhetinic acid, sialic acid, cytotoxicity

## Abstract

Chitosan is the only cationic polysaccharide found in nature. It has broad application prospects in biomaterials, but its application is limited due to its poor solubility in water. A novel chitosan derivative was synthesized by amidation of chitosan with 18β-glycyrrhetinic acid and sialic acid. The chitosan derivatives were characterized by Fourier transform infrared spectroscopy, thermogravimetric analysis, and measurement of the zeta potential. We also investigated the solubility, cytotoxicity, and blood compatibility of chitosan derivatives. 18β-glycyrrhetinic acid and sialic acid could be grafted onto chitosan molecular chains. The thermal stability of the synthesized chitosan derivatives was decreased and the surface was positively charged in water and phosphate-buffered saline. After chitosan had been modified by 18 β-glycyrrhetinic acid and sialic acid, the solubility of chitosan was improved greatly in water and phosphate-buffered saline, and percent hemolysis was <5%. Novel amphiphilic chitosan derivatives could be suitable polymers for biomedical purposes.

## 1. Introduction

18β-Glycyrrhetinic acid (GA) is released from glycyrrhizin through hydrolysis. It is extracted from the roots of licorice, and exhibits various anti-inflammatory [1,2,3,4,5], antioxidation [6,7,8,9], and antimicrobial activities [10]. GA is used widely in the pharmaceutical industry [11]. In particular, due to its anti-inflammatory and antiallergic activities, it is used against skin disorders, such as treatment of atopic dermatitis, pruritus, and acne vulgaris [12,13,14,15]. Recent studies [16,17] have shown that GA has an antiphotoaging effect, which could expand its potential application in the cosmetic field [18,19,20]. However, GA is poorly soluble in water with low bioavailability, and long-term use can cause hyperaldosteronism: these factors limit its application greatly. To improve the activity of GA, the structure has been modified, and good results have been achieved [21,22,23,24,25]. Simultaneously, it has been found that GA can target hepatocyte membranes [26]. Some studies have shown that GA, upon insertion into the molecular chains of chitosan (CS), acts as a targeted carrier to deliver drugs to the liver for therapeutic purposes [27,28,29].

The monosaccharide derivative sialic acid (SA) is a receptor of influenza viruses, and can prevent bacteria from invading cells. Researchers have studied the effect of SA on the immune response [30,31], anti-inflammatory actions [32,33,34,35], antibacterial actions [36], antitumor effects [37], and drug delivery [38]. SA is distributed widely in animals and microorganisms in the form of monomers and polymers [39]. Sialylation can enhance the anti-inflammatory activity of the immunoglobulin (Ig)G antibody and promote the escape of microorganisms from the host immune system [39]. These phenomena have made sialylation a research hotspot.

As the only cationic polysaccharide found in nature, CS has several advantages: it is nontoxic, biodegradable, and has good biocompatibility and antibacterial properties. In recent decades, it has been studied widely and deeply as a biomedical material [40]. However, its application is limited due to its many intramolecular and intermolecular hydrogen bonds, which result in the poor solubility of CS in water [41]. Several studies [42,43,44] have been carried out to improve the water solubility of CS by modifying its molecular structure to break the hydrogen bonds within or between CS molecules. In many of these modification studies, amphiphilic CS has been an important class of CS derivative. Hydrophobic and hydrophilic groups are introduced into the molecular structure of CS, and nanoscale micelles can be obtained through molecular self-assembly [45,46,47,48,49]. These types of nanoscale micelles can embed fat-soluble active substances, which improves the application range of drugs with poor solubility in water. However, few studies have involved the “grafting” of fat-soluble drugs as hydrophobic groups into CS molecular chains. This may be because the activity of fat-soluble drugs decreases (or is even lost) after grafting onto CS molecular chains, which is unfavorable for their application. Conversely, the biological activity of a drug may be unchanged (or its activity weakened only slightly) after a drug molecule is grafted onto CS molecular chains. Meanwhile, CS itself has certain biological activity, which may engender the modified product with new biological activities or synergistic effects upon modification of CS molecular chains. For example, despite being an efficacious antibacterial drug, gentamicin has been used rarely clinically because of serious drug resistance and obvious side-effects. However, the antibacterial activity of gentamicin was not weakened when it was introduced into CS molecular chains by Yan and colleagues [50]. That finding could enable gentamicin to be developed in aquaculture [51]. Protocatechuic acid, as an active substance, has antioxidant, antitumor, and neuroprotective effects. Xu et al. [52] grafted protocatechol onto carboxymethyl chitosan by *N*-(3-Dimethylaminopropyl)-*N*’-ethylcarbodiimide hydrochloride/*N*-hydroxysuccinimide (EDC/NHS) reaction to improve its antioxidant activity. It can be used as a promising antioxidant material for drug delivery and tissue engineering. Based on chitosan, Fathi et al. [53] designed methotrexate-conjugated multifunctional nanoparticles for targeted delivery of erlotinib, which has potential application in targeted therapy of ovarian cancer.

In the present study, GA and SA were incorporated into CS molecular chains to prepare an amphiphilic type of CS, which was then characterized. The cytotoxicity of the CS derivative was investigated using a human keratinocyte (HaCaT) line. Due to the introduction of active substances, it is expected that the new CS derivative could be applied in the treatment of skin burns/scalds, exogenous skin diseases, transdermal delivery of drugs, and cosmetics.

## 2. Materials and Methods

### 2.1. Materials

CS (molecular weight = 100 kDa, degree of deacetylation >95%) was purchased from Cool Chemistry (Beijing, China). GA (purity >98%) was obtained from Gansu Fanzhi Pharmaceuticals (Gansu, China). SA (purity >98%), *N*-Hydroxysuccinimide (NHS; purity >98%), and *N*-(3-Dimethylaminopropyl)-*N*’-ethylcarbodiimide hydrochloride (EDC; purity >98%) were purchased from Aladdin (Shanghai, China). Acetic acid and other chemical reagents were from Shanghai Macklin Biochemicals (Shanghai, China). Cell culture medium (Dulbecco’s modified Eagle’s medium), fetal bovine serum (FBS), and trypsin were from Gibco (Billings, MT, USA). HaCaT cells were supplied by Guangzhou University of Chinese Medicine (Guangzhou, China).

### 2.2. Synthesis of Chitosan-18β-Glycyrrhetinic Acid (CS-GA) and Chitosan-Sialic Acid (CS-SA)

The synthesis of CS-SA and CS-GA was based on a protocol described previously [35,54] with some modifications. First, 1 g of CS powder was dissolved in 50 mL of 0.1 M hydrochloric acid to obtain a homogeneous CS solution (20 mg/mL). Meanwhile, GA (100 mg) was dissolved in 50 mL of anhydrous ethanol. Then, equal amounts of EDC and NHS (mass ratio, EDC/NHS:GA = 1.2:1) were added to the GA solution to activate the carboxyl group of GA at 60 °C and pH 5.0–6.0 (adjusted by PBS) for 30 min. Next, 50 mL of activated GA solution was added dropwise into preheated (60 °C) CS solution under continuous stirring for 24 h. Finally, the synthetic mixture solution was dialyzed against distilled water using a dialysis bag (molecular weight cutoff: 3.5 kDa; Viskase, Willowbrook, IL, USA) for 3 days to remove the remaining water-soluble reagents, and then lyophilized to obtain the CS-GA product. The product, which contained GA with unreacted components, was washed several times with absolute ethanol. Then, it was dried at 60 °C to obtain the final CS-GA conjugate. The synthesis method of CS-SA was consistent with that of CS-GA, the difference was that the CS-SA product could be obtained after freeze-drying without washing with ethanol.

### 2.3. Synthesis of Sialic Acid-Chitosan-18β-Glycyrrhetinic Acid Conjugates (SA-CS-GA)

First, CS-GA (1 g) was dissolved in 50 mL of distilled water to obtain a CS-GA solution. Meanwhile, SA (100 mg) was dissolved in 50 mL of anhydrous ethanol. Then, equal amounts of EDC and NHS (mass ratio, EDC/NHS:GA = 1.2:1) were added to the SA solution to activate the carboxyl group of SA at 60 °C and pH 5.0–6.0 (adjusted by PBS) for 30 min. Next, activated SA solution (50 mL) was added dropwise into preheated (60 °C) CS-GA solution under continuous stirring for 24 h. Finally, the synthetic mixture solution was dialyzed against distilled water using a dialysis bag (molecular weight cutoff: 3.5 kDa) for 3 days to remove the remaining water-soluble reagents and unreacted SA, and then lyophilized to obtain SA-CS-GA conjugates.

### 2.4. Solubility Test

The solubility test of CS derivatives in different solvents was based on the literature [43,44] with some modifications. Briefly, a sample (0.1000 g) was added to 5.00 mL of solvent (water or phosphate-buffered saline (PBS)) and stirred overnight at 25 °C to prepare a saturated solution with the insoluble part suspended in solution. After centrifuging the saturated solution (5780× *g*, 10 min, room temperature), the residual solid was washed thrice with acetone and dried in a vacuum drying oven for 24 h and finally weighed. The solubility was calculated as shown below:

(1) $$Solubility=\frac{0.1000-W}{5.00}$$

where *W* is the weight of the vacuum-dried residual solid (g).

### 2.5. Fourier Transform Infrared (FTIR) Spectroscopy

The sample was prepared using potassium–bromide pellets. The scanning range was 400 cm^−1^ to 4000 cm^−1^ with a resolution of 4 cm^−1^. Briefly, under an infrared lamp, the sample (0.20–1.50 mg) and potassium bromide (spectral grade, 200–300 mg) were placed in an agate mortar, mixed and ground for 2–3 min. After being exposed to a pressure of 20 MPa for 1–2 min, a 13-mm pellet was obtained.

### 2.6. Thermogravimetric Analysis (TGA)

TGA and differential thermogravimetric analysis (DTA) were undertaken with a TGA 5500 analyzer (TA Instruments, New Castle, DW, USA). Each sample weighed 2.0–2.5 mg. TGA was done between 30 °C and 600 °C at a heating rate of 10 °C/min in a nitrogen atmosphere.

### 2.7. Measurement of the Zeta Potential (ZP)

To determine the charge of the novel CS derivative, the ZP of the polymer was measured using Zetasizer™ Pro (Malvern Instruments, Malvern, UK). The voltage applied to the driving electrodes of the capillary electrophoresis cell was 150 V. The capillary cell (DTS1070; Malvern Instruments) was rinsed with ultrapure water to ensure the stability of the measurements before each use. All experiments were conducted at 25 °C. The measurement was repeated thrice, and values presented as the mean ± standard deviation (SD).

### 2.8. Cytotoxicity Test

The cytotoxicity of the new CS derivatives was evaluated by the Cell Counting Kit (CCK)-8 test. HaCaT cells were seeded into 96-well plates at 8000 cells/well. After 24 h, the culture medium was aspirated and replaced by 100 μL of fresh medium. Then, 10 μL of CS derivatives of different concentrations (dissolved in PBS at pH 7.2) were added and cultured in a cell incubator for 24 h. CCK-8 (10 μL) was added into each well, and then the absorbance was determined at 450 nm after 2-h incubation. Cell viability was calculated as the ratio of absorbance of wells with polymer treatment to that of untreated wells.

### 2.9. Hemolysis Test

The hemolytic activity of CS derivatives was investigated according to the method described by Fisher and colleagues [55]. Briefly, 0.5 mL of CS derivatives of different concentrations (dissolved in PBS at pH 7.2) were mixed and shaken gently with 2% red blood cells (RBCs; 0.5 mL) and then incubated in a water bath at 37 °C for 1 h. Subsequently, they were centrifuged at 1000 rpm for 5 min at room temperature. Next, 100 μL was removed and placed in 96-well plates. The absorbance was detected at 540 nm with a microplate reader (DNM-9602; Perlong, Beijing, China). Water and PBS were used as positive and negative controls, respectively. Three parallels were set for each sample. Percent hemolysis was calculated using the following formula:

(2) $$Percent\;hemolysis=\frac{A_{s}-A_{n}}{A_{p}-A_{n}}\times 100\%$$

where *A_S_* is the absorption value of the sample, *A_n_* is the absorption value of the negative control, and *A_p_* is the absorption value of the positive control.

### 2.10. Statistical Analyses

Data are presented as the mean ± standard deviation (SD). Statistical analyses were undertaken by the Student’s *t*-test using the statistical software Prism 8.0 (GraphPad, San Diego, CA, USA). *p* < 0.05 was considered statistically significant.

## 3. Results and Discussion

### 3.1. Synthesis of Novel CS Derivatives

**Figure 1:**
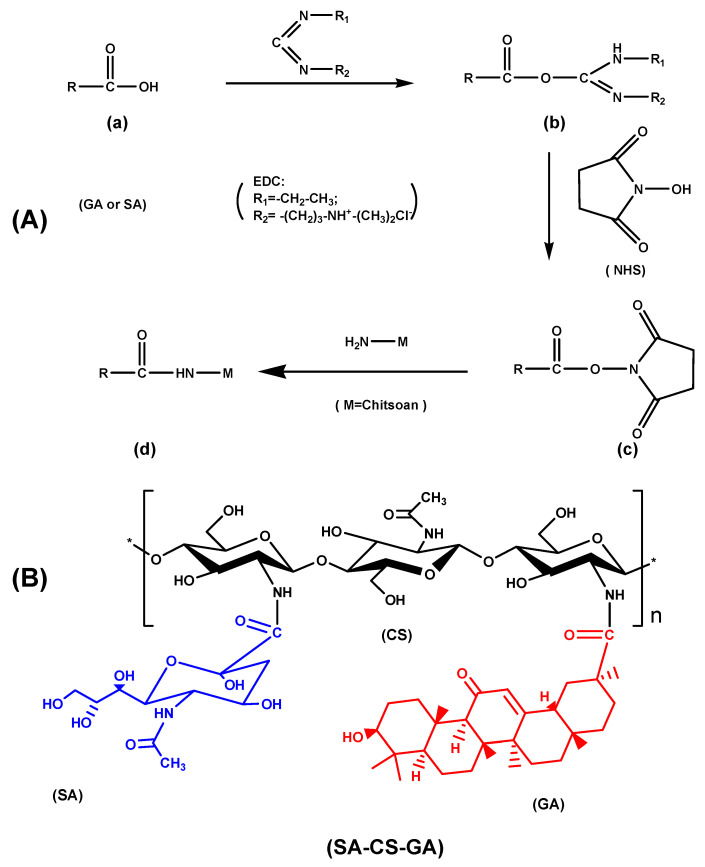
Graft mechanism of chitosan (CS) and 18β-glycyrrhetinic acid (GA) or sialic acid (SA) in the presence of *N*-(3-Dimethylaminopropyl)-*N*’-ethylcarbodiimide hydrochloride/*N*-hydroxysuccinimide (EDC/NHS) (**A**); molecular structure of amphiphilic chitosan (**B**).

EDC/NHS chemistry is used extensively to couple two proteins, haptens to carrier proteins, surface molecule attachment and a host of other applications. Practically, any two molecules having a carboxyl group and amine group can be conjugated by this chemistry. The EDC/NHS coupling reaction is dependent upon temperature, pH, and the steric effect [56]. The optimum reaction conditions for our synthesis were pH between 5.0 and 6.0 and temperature at 60 °C below the boiling point of ethanol. In the presence of EDC/NHS, the amino group on CS molecular chains reacts with the carboxyl group on GA or sialic acid SA, and is exhibited in Figure 1A. First, the carboxyl group of GA or SA (a) reacts with EDC to form intermediate (b). The latter reacts with NHS to obtain the NHS active ester (c). Then, (c) reacts with the primary amino groups of CS to obtain CS-GA or CS-SA (d). Additionally, GA and SA can be grafted onto CS molecular chains by amidation to obtain amphiphilic CS (Figure 1B).

### 3.2. Solubility of Novel CS Derivatives

The solubility of CS derivatives in deionized water and PBS at different pH is shown in Table 1. Amphiphilic CS derivatives had good solubility in water and PBS at different pH (>20 mg/mL). GA- or SA-grafted CS derivatives also had good solubility in deionized water (>20 mg/mL), but the solubility in PBS was significantly lower than that in deionized water; nevertheless, the solubility in PBS at pH = 4.0 was higher than that in PBS at pH = 7.2. Hence, the solubility of GA- or SA-grafted CS derivatives was improved compared with that of CS, which was not soluble in water and slightly soluble in acidic PBS. After grafting of GA and SA onto the molecular chains of CS, the solubility in water was improved greatly. Also, the solubility in PBS was significantly higher than that of GA- or SA-grafted CS derivatives.

### 3.3. FTIR Spectroscopy of Novel CS Derivatives

**Figure 2:**
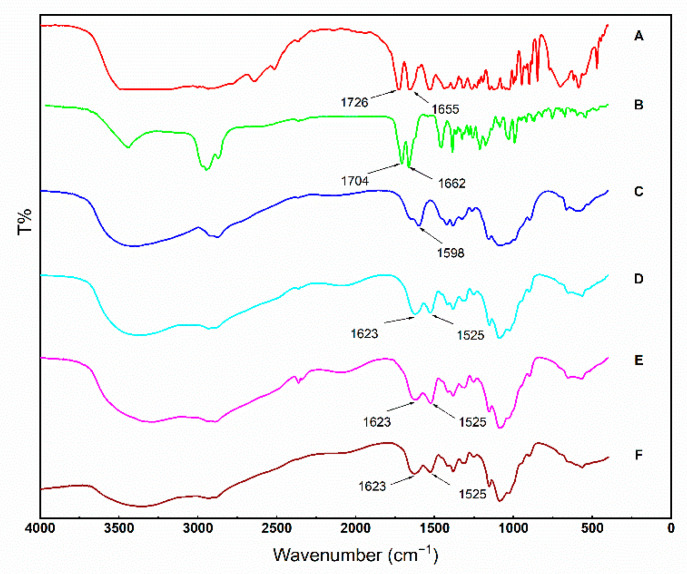
FTIR spectra of sialic acid (**A**), glycyrrhetinic acid (**B**), chitosan (**C**), sialic acid-grafted chitosan (**D**), glycyrrhetinic acid-grafted chitosan (**E**), sialic acid- and glycyrrhetinic acid-grafted chitosan (**F**).

To verify that SA and GA had been introduced into CS chains, FTIR spectroscopy of SA, GA, CS, SA-CS, CS-GA, and SA-CS-GA was employed to characterize intermolecular conformational changes. The characteristic absorption bands of SA (Figure 2A) were 1725 and 1655 cm^−1^, which represented the stretching vibration of the carbonyl group in carboxyl groups and amide groups, respectively. However, in the FTIR spectra of GA (Figure 2B), the characteristic absorption bands associated with the stretching vibration of the carbonyl group in carboxyl groups and ketone groups appeared at 1704 and 1664 cm^−1^, respectively. CS displayed a weak absorption peak at 1655 cm^−1^ (amide I band) and a relatively strong absorption peak at 1598 cm^−1^ (amide II band), which was attributed to the plentiful free amine groups in CS (Figure 2C). In contrast with CS, all of the grafted products, CS-SA (Figure 2D), CS-GA (Figure 2E), and SA-CS-GA (Figure 2F) exhibited an enhanced absorption peak at 1623 cm^−1^ (amide I) and the absorption peak of amide II appeared at 1525 cm^−1^. Simultaneously, the absorption bands at 1725 and 1704 cm^−1^ derived from the carboxyl groups of SA and GA disappeared. Furthermore, the absorption peaks of amide I and amide II of all CS-grafted products strengthened and shifted slightly to lower wavenumbers. These phenomena were consistent with results from other studies [54,57]. The changes in the FTIR spectra of all CS-grafted products suggested that SA and GA were conjugated to the molecular chains of CS by the formation of amide linkages.

### 3.4. Thermal Stability of Novel CS Derivatives

**Figure 3:**
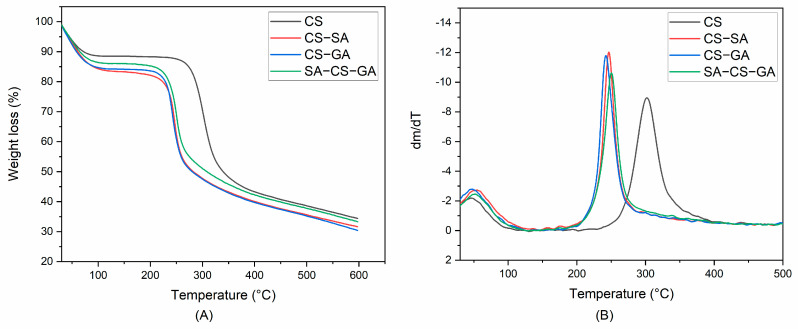
Thermogravimetric (TG) (**A**) and differential thermogravimetric analysis (DTA) (**B**) curves of chitosan (CS), chitosan-sialic acid (CS-SA), chitosan-18β-glycyrrhetinic acid (CS-GA), and sialic acid-chitosan-18β-glycyrrhetinic acid (SA-CS-GA).

Water-absorption and degradation behavior of polymers can be evaluated by TGA [58]. Thermal degradation behavior of CS and its novel derivatives displayed two stages upon heating from 30 °C to 600 °C (Figure 3A). The first stage was the weight loss of bound water and unbound water of CS molecular chains. The content of bound water and unbound water in CS (11.6%) was lower than that in GA- or SA-grafted CS derivatives and amphiphilic CS derivatives (CS-SA, 16.7%; CS-GA, 15.9%; SA-CS-GA, 14.0%) (Figure 3A). This phenomenon was due to the stronger force of hydrogen bonding within or between the molecules of CS, which reduced the content of bound water and unbound water. However, after modification by hydrophobic and hydrophilic groups, the hydrogen bond of CS molecules was destroyed and, thus, it was easier to absorb water. The DTA curves of CS and its derivatives are shown in Figure 3B. The temperature corresponding to the maximum weight loss of CS and CS-GA was similar (48.0 and 47.2 °C, respectively) (Table 2). However, the temperature corresponding to the maximum weight loss of CS-SA and SA-CS-GA increased to 53.8 and 51.8 °C, respectively. The possible reason for this finding was that the content of bound water increased with the increase in the number of hydrophilic groups, and decreased with the increase in the number of hydrophobic groups.

The second stage was the degradation of CS molecular chains. Degradation of CS and its derivatives took one step (Figure 3). The characteristic temperatures of the degradation reaction are given in Table 2, where T_0_ is the temperature at which degradation starts, T_p_ is the temperature at which maximum degradation occurs, and T_f_ is the temperature at which degradation is complete. Compared with CS, the characteristic temperatures of CS derivatives stated above were reduced to a certain extent. However, the characteristic degradation temperatures of CS-GA were slightly lower than that corresponding to CS-SA. The T_0_ of SA-CS-GA was between the T_0_ of GA- or SA-grafted CS derivatives. However, the T_p_ (250.3 °C) and T_f_ (270.1 °C) of SA-CS-GA was higher than those of GA- or SA-grafted CS. These results indicated that GA and SA had been grafted onto CS molecular chains, and resulted in a decrease in the thermal stability of CS.

### 3.5. ZP of Novel CS Derivatives

ZP is related to the electrokinetic behavior of the material molecules [59]. The ZP of CS and three CS derivatives in PBS of different pH is shown in Table 3. The ZP of CS and its derivatives was positive at pH 4.0, indicating that the surface of CS and CS derivatives was positively charged (which is caused by the primary amino group on CS molecular chains). At pH 7.2, negative ZP of CS was determined, which was caused by deprotonation. At pH 4.0, the ZP of CS was higher than that of modified CS, indicating that the number of primary amino residues in modified CS decreased, which demonstrated (indirectly) the amidation reaction. The ZP of the three CS derivatives at pH = 4.0 was higher than that at pH = 7.2, which was due to protonation of the primary amino group. The lower the pH, the higher the ZP [58,60], and our results were consistent with this general rule.

### 3.6. Cytotoxicity of Novel CS Derivatives

**Figure 4:**
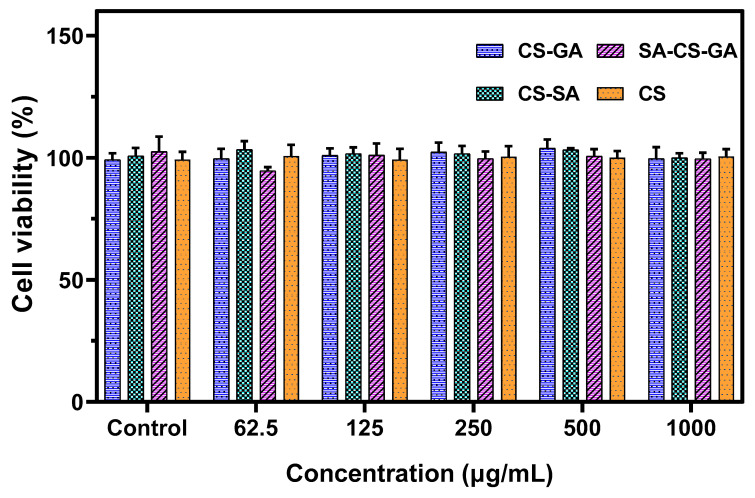
Viability of HaCaT cells after 24-h exposure to novel chitosan derivatives at different concentrations. Data are presented as mean ± SD.

The cytotoxicity and blood compatibility of materials are essential indices for application in biological fields [59]. The toxicity of novel CS derivatives against HaCaT cells is shown in Figure 4. The viability of HaCaT cells treated with different concentrations of CS-GA, CS-SA, and SA-CS-GA for 24 h was not significantly different compared with that in the control group. These results showed that CS derivatives had no toxic or side effects on HaCaT cells.

**Figure 5:**
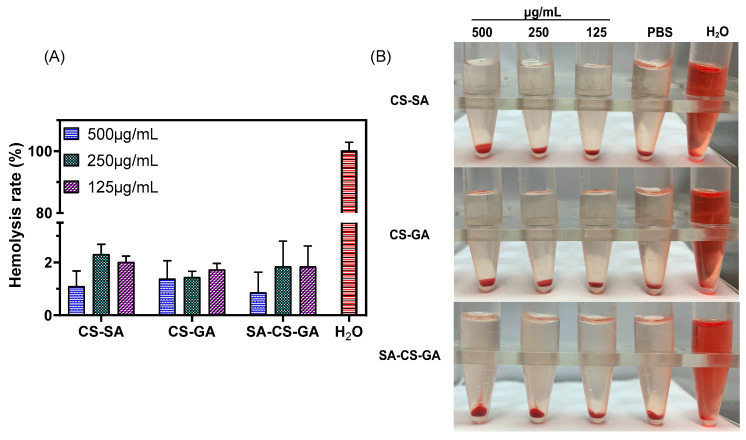
Hemocompatibility of novel chitosan derivatives at different concentrations. (**A**), hemolysis rate; (**B**), hemolysis photograph. Data are presented as mean ± SD of three independent experiments.

A hemolysis test can be used to evaluate the blood compatibility of biomaterials [61]. Percent hemolysis <5% meets the requirements for application of biological materials in medicine. Evaluation of blood compatibility of CS derivatives of different concentrations is shown in Figure 5. Significantly different from water-treated RBCs, no obvious rupture of RBCs was found in samples treated with CS-GA, CS-SA, and SA-CS-GA. Similar to samples treated by PBS, percent hemolysis was <5% within the concentration range, and there was no significant difference between the groups of modified CS derivatives. These results showed that the modified CS did not damage RBCs when the concentration was <500 μg/mL. In addition, compared with CS-GA and CS-SA, SA-CS-GA had lower percent hemolysis at a higher concentration and better blood compatibility.

## 4. Conclusions

GA and SA were grafted onto CS molecular chains by amidation. Compared with pure CS, due to the destruction of intramolecular and intermolecular hydrogen bonds, the thermal stability of CS derivatives decreased, and the solubility in water and PBS was increased considerably. The unreacted primary amino groups in the CS molecular chains made the CS derivatives’ surface positively charged, and the ZP of amphiphilic chitosan was lower than that of others. The novel CS derivatives we synthesized were not toxic to keratinocytes and had good blood compatibility. The synthesized CS could be applied for skin repair and targeted delivery of drugs.

## Figures and Tables

**Table 1:** Solubility of novel chitosan derivatives. Data are presented as mean ± SD of three independent experiments.

Sample	Solubility (mg/mL)		
	Water	PBS (pH = 7.2)	PBS (pH = 4.0)
CS	0	0	1.36 ± 0.25
CS-SA	>20.00	5.97 ± 0.32	12.39 ± 0.30
CS-GA	>20.00	5.65 ± 0.37	9.72 ± 0.28
SA-CS-GA	>20.00	>20.00	>20.00

**Table 2:** Characteristic temperatures of thermal degradation of CS and its derivatives.

Samples	First Stage	Second Stage		
	T_p_/°C	T_0_/°C	T_p_/°C	T_f_/°C
CS	48.0	279.9	301.9	327.9
CS-SA	53.8	235.1	246.4	261.7
CS-GA	47.2	231.6	242.2	259.5
SA-CS-GA	51.8	234.0	250.3	270.1

**Table 3:** Zeta potential of novel chitosan derivatives at pH = 4.0 and pH = 7.2 in PBS. Data are presented as mean ± SD of 3 independent experiments.

Sample	Zeta Potential (mV)	
	pH = 4.0	pH = 7.2
CS	28.40 ± 1.13	−3.59 ± 0.25
CS-SA	24.41 ± 1.69	8.12 ± 0.86
CS-GA	22.12 ± 0.54	8.64 ± 1.56
SA-CS-GA	8.34 ± 0.84	4.13 ± 0.87

## Data Availability

The data presented in this study are available on request from the corresponding author.

## Sample Availability

Samples of the compounds are not available from the authors.
